# Temporal dynamics of emotional face processing in social anxiety

**DOI:** 10.1371/journal.pone.0337403

**Published:** 2025-11-19

**Authors:** Ya-Chun Feng, Bo-Cheng Kuo, Wen-Yau Hsu

**Affiliations:** 1 Institute of Education, National Sun Yat-sen University, Kaohsiung, Taiwan; 2 Department of Psychology, National Chengchi University, Taipei, Taiwan; 3 Department of Psychology, National Taiwan University, Taipei, Taiwan; Nanjing University, CHINA

## Abstract

Previous studies have demonstrated that emotional facial expressions influence attention and perception in individuals with social anxiety. However, the relative influence of positive versus negative expressions on distinct subprocesses of attention and perception remains unclear. This event-related potential (ERP) study investigates the temporal dynamics of electrophysiological responses to emotional faces in high (HSA; N = 56) or low (LSA; N = 47) social anxiety individuals using a dot-probe task. Four face pairs (angry-neutral, happy-neutral, angry-happy, and neutral-neutral) were presented to probe the influence of positive and negative expressions. While behavioural results showed no significant group differences in attention bias, ERP results showed a reduced N170 amplitude for the HSA vs. LSA group in angry-neutral, happy-neutral, and angry-happy face pairs. Furthermore, enhanced N2pc effects to emotional expressions were found only in the HSA group when angry-neutral and happy-neutral face pairs were presented. No N2pc effect emerged when both positive and negative expressions were presented simultaneously. Finally, no significant P1 effect was found. Together, both positive and negative expressions influenced attentional deployment and face-specific processing in relation to social anxiety. Socially anxious individuals perceived less emotional facial information, yet their attention was biased by both negative and positive expressions.

## Introduction

Social anxiety disorder is commonly characterised by the subjective experience of excessive fear in social situations involving scrutiny and potential negative evaluations by others [1]. Individuals with social anxiety often become hypervigilant to cues associated with social evaluation [2,3]. Previous research has demonstrated that emotional facial expressions influence attention and perception in socially anxious individuals [4–7]. However, it remains unclear whether positive expressions can bias attentional and perceptual processing in anxious individuals to the same extent as negative expressions. This study addresses this issue by examining the temporal dynamics of electrophysiological responses to both negative and positive facial expressions in individuals with either high or low social anxiety, using a dot-probe task with electroencephalography (EEG).

Facial expressions are among the most salient forms of social information, providing critical cues about others’ emotions and evaluations. According to the social-evaluative threat principle [8], such information may be perceived as threatening regardless of valence (e.g., angry or happy). For socially anxious individuals, both negative and positive expressions can signal potential judgment and thus be experienced as threatening. Facial expressions also exert a significant influence on attentional and perceptual processes in socially anxious individuals. Previous studies have shown that they may allocate disproportionate attention to socially threatening information, a phenomenon referred to as negative attention bias [8–10]. Accumulating evidence further suggests a comparable bias towards positive faces, described as positive attention bias [5,11]. Although results are mixed when positive and negative expressions are presented simultaneously [12], these observations suggest that socially anxious individuals may perceive both happy and angry faces as evaluative and potentially threatening [13–15].

Although previous evidence has revealed the importance of attention bias towards socially evaluative stimuli in social anxiety [4,8–10], other hypotheses argue that socially anxious individuals may focus on bodily sensations and internal thoughts [16], thereby avoiding the processing of social information [9]. This self-focused attention and avoidance impair the processing of external stimuli, as reflected in poor recognition of neutral faces in memory tasks and difficulty identifying emotionally negative expressions [17,18]. These findings suggest that social anxiety is associated not only with attention bias towards external information but also with heightened internal focus and diminished processing of facial-specific information.

To investigate these attentional and perceptual processes in social anxiety, researchers have widely employed the dot-probe task [19]. In this paradigm, participants respond to probes that follow pairs of stimuli (e.g., faces or words), allowing inferences about attentional allocation towards emotional versus neutral information. Many studies using the dot-probe task have shown that socially anxious individuals tend to respond faster to probes appearing at the same location as emotionally negative stimuli compared to neutral ones [4–7,10]. Although the behavioural effects of the dot-probe task are subject to debate, these findings suggest that socially anxious individuals may allocate attention more towards negative information than neutral one.

While most of the studies have focused on negative attention bias in social anxiety, it remains unclear whether positive expressions can also bias attention to the same extent as negative expressions in highly anxious individuals. In this study, we aim to investigate the influence of emotional facial expressions on temporal dynamics in a dot-probe task for healthy participants with high and low social anxiety. We capitalise on event-related potentials (ERPs), specifically the P1, N170, and N2pc, to investigate the temporal profiles of early sensory processing, face-specific processing and attentional deployment. First, we measured the P1 amplitudes to test whether emotional expressions affect the early processing of face stimuli. The P1 at lateral occipital electrodes indexes the early, stimulus-driven sensory processing approximately 100 ms after stimulus onset [20]. In addition, we measured the N170 amplitudes to test whether emotional expressions affect the perceptual processing of face-specific information. The N170, which peaks around 130–200 ms after stimulus onset over occipitotemporal electrode sites, is sensitive to face perception [21,22]. Previous evidence has shown reduced N170 amplitudes in those with high social anxiety compared to those with low social anxiety [23–25], indicating diminished processing of face-specific information in socially anxious individuals. Finally, we examined the magnitude of the N2pc to test whether emotional expressions elicit stronger attentional deployment for negative or positive faces compared to neutral ones. the N2pc is typically observed over posterior brain regions between approximately 200 and 400 ms after stimulus onset, characterised by greater negative potentials at electrodes contralateral relative to ipsilateral to the attended hemifield, and is considered an established marker of top-down attentional deployment [26,27]. ERP studies have shown that individuals with high social anxiety exhibit enhanced sensory processing or greater attentional deployment when viewing negative faces compared to neutral ones [4,28,29]. A similar enhanced N2pc effect was observed when socially anxious participants encountered emotional faces (e.g., happy, angry) versus neutral face distractors in a visual search task [30].

We expected that emotional facial expressions would have a stronger influence on early sensory processing (indexed by the P1), face perception (indexed by the N170), and attentional deployment (indexed by the N2pc) in individuals with high social anxiety than those with low social anxiety. If socially anxious participants were sensitive to facial expressions regardless of valence, we expected similar processing at the early sensory stage, reflected by similar P1 responses for emotional and neutral faces. If socially anxious participants also avoided processing faces, we would expect a reduced N170 amplitude for the high social anxiety group relative to the low social anxiety group. Finally, if socially anxious participants exhibited an attentional bias towards emotional expressions, we expected a greater N2pc amplitude for emotional faces (e.g., happy, angry) compared to neutral faces. This ERP study aims to enhance our understanding of how emotional facial expressions affect early sensory processing, attentional deployment, and face perception in individuals with social anxiety, complementing behavioural measures.

## Methods

The study received approval from the National Taiwan University Research Ethics Office (201207HS019), and all participants provided written informed consent.

### Participants

We screened 1,113 undergraduate students at National Chengchi University, Taiwan, using the Social Avoidance and Distress Scale (SAD) and Fear of Negative Evaluation Scale (FNE) questionnaires [31]. Participants with the highest 30% (SAD > 80, FNE > 106) and lowest 30% (SAD < 63, FNE < 88) scores in both questionnaires were designated as the high social anxiety group (HSA) and low social anxiety group (LSA), respectively. For the EEG experiment, 61 HSA and 49 LSA individuals participated. The sample size is based on power 0.8 and alpha 0.05, using effect size from the N2pc index when the angry-neutral faces were presented [25], which indicated 43 participants per group. Additional participants were recruited to account for anticipated exclusions and for our subsequent study. Written informed consent was obtained from all participants before the scanning, and they were financially reimbursed for their time ($500 NTD, approximately $17 USD). Questionnaires were retaken before the experiment. Participants not meeting the criteria for both questionnaires before the experiment (n = 3) or with response accuracy below 80% during the experiment (n = 4) were excluded. The final sample comprised 56 HSA (23 males and 33 females, age range = 18–24 years, mean age = 19.93 years) and 47 LSA (20 males and 27 females, age range = 18–23 years, mean age = 19.70 years) participants with normal or corrected-to-normal vision. Participants were recruited between April 15, 2013 and May 13, 2015. See Supporting Information (S1 File) for participant profiles.

### Measures

#### Self-report questionnaires.

Levels of social anxiety were assessed using the Chinese versions of SAD and FNE [31]. The Cronbach’s α for the Chinese versions were.95 and.96 [32]. The SAD includes 28 items and measures avoidance and anxiety/distress in social situations using a 5-point scale ranging from 1 (totally disagree) to 5 (totally agree). Total scores range from 28 to 140. The FNE includes 30 items and measures the fear of others’ evaluation and the degree of avoiding disapproval or seeking approval on the same 5-point scale. Total scores range from 30 to 150. For both questionnaires, higher scores indicate higher levels of social anxiety.

#### Stimuli.

The stimuli were presented using Presentation software (Neurobehavioral Systems, Inc., CA). We used 54 face images from the Taiwan facial expression database [33]. These images included faces from eight males and eight females, each exhibiting three facial expressions (happy, angry, and neutral). To standardise the stimuli, we removed the hair and backgrounds for each image and converted them to grayscale. To control for low-level visual attributes, we also computed the grayscale luminance and contrast of the images, and found no significant differences across emotional categories [mean luminance: *F*(2, 34) = 0.70, *p* = .50; contrast: *F*(2, 34) = 0.63, *p* = .54]. Each face image subtended a visual angle of approximately 6° (width) × 8° (height). In each face pair, two facial images with different expressions (e.g., angry-neutral) from the same individual were presented equidistantly (approximately 5°) from the centre of the screen horizontally. The probe size was approximately 0.8°. All visual stimuli were presented against a black background, and a central fixation point was maintained throughout the experiment.

#### Task.

Participants performed an EEG-recorded dot-probe task [8] in a dimly illuminated room, positioned 70 cm in front a CRT monitor. Each trial began with a 500 ms fixation cross, followed by a 500 ms display of face pair (one face in each hemifield). After a 150–300 ms jittered interval, a probe (“：” or “‥”) appeared for 200 ms. The stimulus onset asynchrony (SOA) between the face pair and the probe ranged from 650 to 800 ms. Participants indicated its orientation (horizontal/vertical) with left/right mouse buttons. An inter-trial interval ranged from 2,000–2,500 ms.

**Fig 1:**
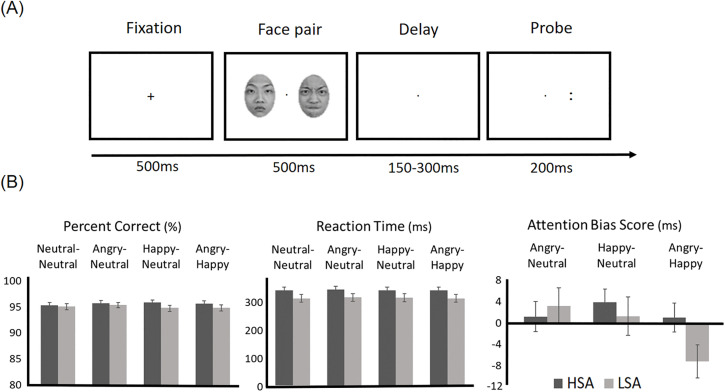
Dot-probe task schematic and behavioural results. **(A)** Each trial began with a centrally displayed fixation cross. Next, a face pair was presented and each face was presented in each hemifield. Following a short delay, a probe was presented and participants indicated its orientation. **(B)** Behavioural results included accuracy (percent correct, %), RT (ms), and attention bias score (ms). Error bars represent standard errors. Asterisk denotes statistical significance.

The task’s schematic is illustrated in Fig 1A. There were four types of face pairs: angry-neutral, happy-neutral, angry-happy, and neutral-neutral. Neutral-neutral face pairs served as a neutral control condition, while angry-neutral and happy-neutral face pairs examined the attention bias for negative and positive expressions in contrast to neutral expression. Moreover, angry-happy face pairs allowed us to directly compare the difference in attentional allocation between negative and positive expressions. Emotional face locations (left and right), probe locations (left and right), and probe orientations (horizontal and vertical) were counterbalanced within participants. The response assignment was also counterbalanced across participants. Half of the participants were instructed to respond “horizontal” by pressing the left mouse button, or “vertical” by pressing the right mouse button. This mapping was reversed for the other half of the participants. Participants were instructed to respond as quickly and accurately as possible. All trial types were equiprobable and randomised within eight blocks of 64 trials, yielding 512 trials in total (128 trials per face-pair condition).

### Behavioural data analysis

Behavioural measures including response time (RT) and accuracy for the dot-probe task were analysed. Only correct responses were included in the RT analyses. Statistical analyses were conducted using IBM SPSS (version 25). We first compared the accuracy differences among four face-pair conditions using a mixed 2 × 4 analysis of variance (ANOVA) with group (HSA, LSA) as a between-group variable and face pair (angry-neutral, happy-neutral, angry-happy, neutral-neutral) as a within-group variable. Additionally, attention bias score (ABS) was computed for each participant by subtracting the mean RT of probes following neutral or happy faces from angry faces for angry-neutral and angry-happy pairs. Negative ABS values indicate attention bias toward angry faces over happy or neutral faces. For happy-neutral pairs, ABS was computed by subtracting the mean RT of probes following neutral faces from those following happy faces. Negative values indicate attention biases towards happy faces over neutral faces. Given that ABS indexed negative and positive attention bias based on different face pairs, three independent *t*-tests were conducted on ABS for each face-pair condition to evaluate attention bias differences between groups.

### EEG recording and processing

The EEG was recorded continuously using NuAmp amplifier (Neuroscan Inc.), from 32 Ag/AgCl electrodes (FP1/FP2, F3/F4, F7/F8, FC3/FC4, FT7/FT8, C3/C4, T3/T4, CP3/CP4, TP7/TP8, P3/P4, T5/T6, O1/O2, FZ, FCZ, CZ, CPZ, PZ, and OZ) placed on the scalp with an elasticated cap, positioned according to the 10–20 international system [34]. Vertical eye movements were recorded by electrodes placed on the supraorbital and infraorbital ridges of the right eye, i.e., vertical electro-oculogram (VEOG). Horizontal eye movements were recorded by electrodes placed on the outer canthi of the right and left eyes, i.e., horizontal electro-oculogram (HEOG). Additional electrodes were used as ground and reference sites. Electrodes were referenced to the right mastoid site (A2) during recording. The electrode between FPZ and FZ (AFZ) on the midline served as the ground electrode. Electrode impedances were kept below 5KΩ. Ongoing brain activity at each electrode site was sampled every 1 ms (1000-Hz analogue-to-digital sampling rate). The activity was filtered with a low-pass filter of 300 Hz and no high-pass filter was used during recording.

Offline, the EEG data were first pre-processed using Neuroscan software. All the EEG data were re-referenced to the algebraic average of the right and left mastoids. Bipolar electro-oculogram (EOG) signals were derived by computing the difference between the voltages of electrodes placed to the side of each eye (HEOG) and above and below the left eye (VEOG). The re-referenced and transformed continuous data were then low-pass filtered (40 Hz) to exclude high-frequency noise. The continuous EEG was then segmented into epochs starting 100 ms before and ending 600 ms after the onset of the face-pair stimulus. The EEG epochs were baseline-corrected with the 100 ms pre-stimulus period. Epochs containing excessive noise or drift (+/-100uV) at any electrode were excluded, and epochs with eye-movement artefacts (blinks or saccades) were also rejected. Blinks were identified as large deflections (+/-50uV) in the HEOG or VEOG electrodes. A visual inspection was also carried out to confirm appropriate removal of artefacts and identify residual saccades in the individual HEOG traces (+/- 10–20 µV). Trials with incorrect behavioural responses were also discarded from the final analyses. A lower limit of 30 artefact-free trials per face condition per subject was set to maintain an acceptable reliability [35].

### ERP analyses

The ERP analyses were conducted using the ERPLAB toolbox in MATLAB [36]. The mean amplitudes of the P1 were calculated with an 80 ms to 120 ms time window at the left and right lateral occipital-temporal electrodes (T5 and T6, which are close to P7 and PO7 and P8 and PO8, respectively) [23]. The mean amplitudes of the N170 were measured within a 160 ms to 180 ms time window at T5/6 and O1/2 electrodes [22,37]. Mean amplitudes of the N2pc were measured with a 200 ms to 260 ms time window at the T5 and T6 posterior electrode pairs contralateral and ipsilateral to the location of the emotional stimuli [38] when face pairs were angry-neutral and happy-neutral. We also computed the N2pc for the angry face as the target for the angry-happy face pair.

We first conducted a mixed 2 × 2 × 4 ANOVA on each P1 and N170 amplitude, with group (HSA, LSA) as a between-group variable, and electrode site (left, right) and face pair (angry-neutral, happy-neutral, angry-happy, and neutral-neutral) as within-group variables. We also conducted three mixed 2 × 2 ANOVAs on the N2pc amplitudes for each emotional face pair with group (HSA, LSA) as a between-group variable, and with electrode site (ipsilateral, contralateral to the location of the angry face for angry-neutral and angry-happy face pair, and happy face for the happy-neutral face pair) as within-group variables. Neutral-neutral face pair was not included in the N2pc analysis because attention allocation cannot be identified when the same stimuli were presented at both locations. Mauchly’s test of sphericity was conducted for all the ANOVA analyses. When the assumption of sphericity was violated, the Greenhouse-Geisser correction was applied to adjust the degrees of freedom. See Supporting Information (S1 File) for all detailed results.

## Results

### Behavioural results

The mixed 2 × 4 ANOVA analyses on accuracy data showed no significant effect of group, face pair, or their interaction (*p*s* *> .37). For the *t*-*t*est analyses of the ABS, none of the comparisons reached statistical significance after applying the Bonferroni correction (*α* = .017). The behavioural results are illustrated in Fig 1B.

### ERP results

#### P1 amplitude.

Analyses of the P1 amplitude showed a significant main effect of electrode site [*F*(1, 101) = 22.28, *p* < .001, *η*^2 ^= .18], indicating a greater P1 amplitude at the right (1.61 ± 0.17 µV) relative to the left (0.72 ± 0.14 µV) electrode site. No other effect was significant (*p*s > .1).

#### N170 amplitude.

**Fig 2:**
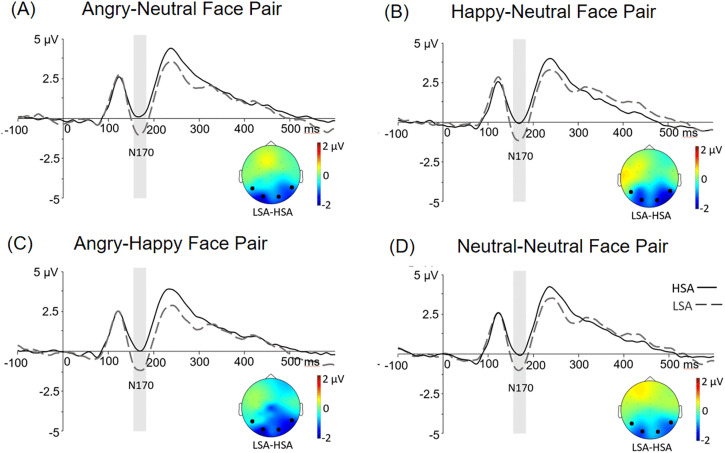
The results of the N170. Grand-mean potentials are presented for (A) angry-neutral, (B) happy-neutral, (C) angry-happy, (D) neutral-neutral face pairs at T5/6 and O1/2 electrodes for the HSA (solid line) and the LSA (dash line) groups. The shaded area represents the N170 time window (160-180ms). The topographical maps present the difference in N170 between the LSA and HSA group in each face pair condition.

The N170 results showed a significant main effect of group [*F*(1, 101) = 4.92, *p* = .029, *η*^2 ^= .05], indicating a greater N170 for the LSA group (−1.02 ± 0.36 µV) than the HSA group (0.08 ± 0.33 µV). Moreover, the interaction among face pair, electrode site, and group was significant [*F*(3, 303) = 2.76, *p* = .042, *η*^2 ^= .03]. Bonferroni-corrected post-hoc comparisons showed greater N170 amplitudes for the LSA group relative to the HSA group at the right hemisphere when the face pairs were happy-neutral (*p* = .028) and angry-happy (*p* = .030). A greater N170 amplitude was also found in the LSA group than the HSA group at the left hemisphere when the face pair was angry-neutral (*p* = .039). No significant effect was obtained for neutral-neutral face pair. The N170 results are illustrated in Fig 2.

#### N2pc amplitude.

Finally, analyses of the N2pc for the angry-neutral face pair showed a significant main effect of electrode site [*F*(1, 101) = 13.06, *p* < .001, *η*^2 ^= .12], reflecting more negative voltage over contralateral electrodes (2.05 ± 0.24 µV) compared to ipsilateral electrodes (2.43 ± 0.23 µV) to the angry face. The main effect of electrode site was also found in the happy-neutral face pair [*F*(1, 101) = 10.24, *p* = .002, *η*^2 ^= .09], reflecting more negative voltage over contralateral electrodes (1.91 ± 0.23 µV) compared to ipsilateral electrodes (2.23 ± 0.22 µV) to the happy face. For the angry-happy face pair analysis, there was a main effect of group [*F*(1, 101) = 3.89, *p* = .051, *η*^2 ^= .04], suggesting a smaller amplitude for the LSA (1.41 ± 0.32 µV) than the HSA group (2.25 ± 0.29 µV). No N2pc effect was significant for this face pair (*p*s > .2).

**Fig 3:**
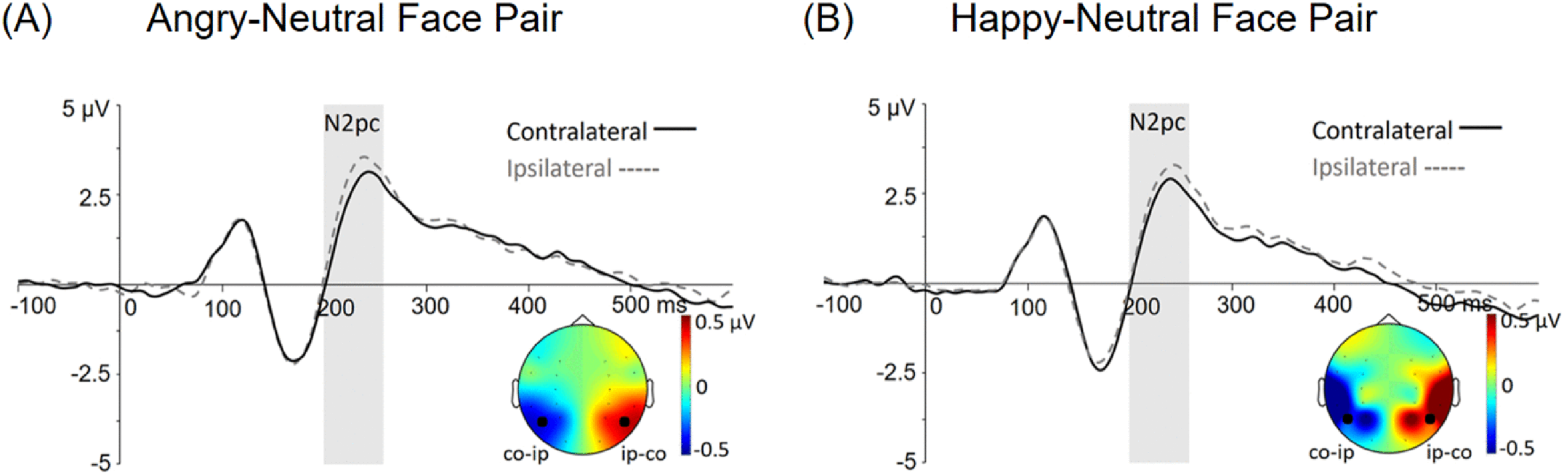
The results of the N2pc in the HSA group. Grand mean potentials are presented for the (A) angry-neutral and (B) happy-neutral face pairs at T5/6 electrodes, showcasing the contralateral (co, thick line) and ipsilateral (ip, dash line) waveforms to emotional faces. The shaded area represents the N2pc time window (200-260ms). Additionally, topographical maps of the N2pc depicted the contralateral minus ipsilateral (co-ip, left/blue), and ipsilateral minus contralateral (ip-co, right/red) difference waves.

To better clarify the effects, we analysed the N2pc effect based on the differential amplitudes between the electrode sites (contralateral versus ipsilateral) for each face pair for each group separately. Bonferroni-corrected comparisons showed significant N2pc effects for angry-neutral (*p* = .002) and happy-neutral face pairs (*p* = .010) for the HSA group. However, this was not true for the angry-happy pair for the HSA group and for all face pairs for the LSA group (*ps* > .057). The N2pc amplitudes are illustrated in Fig 3.

## Discussion

This ERP study investigated the impact of emotional facial expressions on attentional and perceptual processes in individuals with high or low social anxiety. Although we did not find significant effects in the behavioural data or the P1 responses, we observed an enhanced N170 for the LSA group in comparison to the HSA group when emotional faces were presented in angry-neutral face pairs at the right hemisphere, and happy-neutral and angry-happy face pairs at the left hemisphere. Moreover, greater N2pc effects on emotional faces were found in the HSA group only when angry-neutral and happy-neutral face pairs were presented. However, no significant N2pc effect was observed for the LSA group. Together, our results showed that emotional facial expressions influenced face-specific processing and attentional deployment, as indexed by the N170 and the N2pc, in relation to social anxiety.

Some studies have shown that social anxiety levels are associated with facial perception, reflected by smaller N170 amplitudes in the social anxiety group than the control group [23–25]. Consistent with these findings, we found a stronger reduction in N170 amplitude in the HSA group than the LSA group only when emotional faces were presented. Our N170 result suggests that individuals with high social anxiety perceived less information from emotional expressions than those with low social anxiety, and this was not true when they perceived neutral faces. This result also reflects that socially anxious individuals may avoid processing emotional faces.

The reduced facial processing, as reflected in the N170, is consistent with predictions from the self-focus account of social anxiety [16]. The cortical sources generating the N170 (or its magnetic counterpart, the M170) have been localised to the inferior occipital and fusiform gyri using magnetoencephalography (MEG), and are associated with face encoding and recognition [39]. Specifically, reduced N170 amplitudes suggest that HSA individuals tend to allocate more resources to internal information rather than external inputs when perceiving social cues such as emotional faces. This interpretation also aligns with the concept of cognitive avoidance, a core feature of social anxiety characterised by the tendency to avoid negative thoughts, feelings, or physical sensations [8,9,40]. Although avoidance behaviours may provide temporary relief from anxiety triggers, they can also prevent individuals with social anxiety from actively participating in social interactions. As a result, such individuals may struggle to derive positive experiences from social encounters and to re-evaluate information initially perceived as threatening. Consequently, their false evaluations of social information may persist, thereby maintaining anxiety. An alternative interpretation of the reduced N170 is that heightened vigilance consumes early attentional resources, leaving fewer resources available for facial processing in socially anxious individuals. However, because we did not observe any significant influence on early sensory processing (as reflected in the P1), the N170 findings cannot be conclusively explained by the early attentional resource account. In summary, the reduction in N170 amplitudes for emotional faces observed in our study supports the roles of self-focusing and cognitive avoidance as key characteristics of social anxiety.

In addition to the N170, the N2pc effect indicated the deployment of attention towards emotional information within the face pairs. The N2pc is closely associated with the deployment of attention towards targets during visual search [26,41] and this effect has been localised to neural generators in the posterior parietal and occipital regions [42]. Previous studies have demonstrated that threatening faces elicit an enhanced N2pc in individuals with high trait anxiety levels [28]. However, some studies did not find a relationship between the N2pc and anxiety [43,44]. The current study extends these findings by including different combinations of emotional expressions. Our N2pc results indicate that the HSA group exhibited a robust bias towards emotional expressions, particularly when angry-neutral or happy-neutral face pairs were presented. These results provide strong support for the social-evaluation threat principle [8], which suggests that social-evaluation information can be threatening for socially anxious individuals, regardless of its emotional content. For example, a smiling face or a compliment can be perceived as threatening, as they may indicate an expectation for high performance in the future, which is associated with unachievable challenges or potential future failures [15]. Moreover, the absence of a significant N2pc effect when both negative and positive expressions were presented simultaneously also supports this perspective. In this case, both emotional expressions may simultaneously capture socially anxious individuals’ attention. We found that the LSA group showed a stronger negative bias than the HSA group when both emotional faces were presented. These results indicate that negative stimuli may bias attention over positive stimuli for the general population. Together, these results showed that socially anxious individuals may tend to avoid processing emotional facial information. However, their attention is still drawn to both positive and negative faces over neutral faces because emotional expressions may be perceived as threatening to them. These ERP results provide valuable insights into how social anxiety affects attention allocation and emotional processing.

Our analysis regarding the P1 amplitude did not reveal any significant effect between groups or among the face pairs. It is worth noting that the literature on P1 amplitude using the dot-probe task has yielded mixed results. For example, a study found enhanced P1 amplitudes for the angry-neutral face pair compared with the happy-neutral pair in the HSA group; however, they did not observe any difference in P1 amplitude between the HSA and LSA groups [23]. In contrast, some studies have shown an enhanced P1 for the HSA group compared to the LSA group [4,29]. We speculate that the discrepancy in the P1 may result from variations in stimulus encoding across different tasks. Specifically, studies that reported significant group differences in P1 amplitude included elements such as priming words presented before faces [4], or had more trials with target probes following threatening faces than neutral faces [29]. Additionally, factors such as the threatening level of stimuli, the severity of social anxiety in the participants, and the task demands [45] may also contribute to the inconsistent findings in P1 amplitude when comparing the HSA with the LSA group.

While some studies have linked social anxiety to negative attention bias using behavioural measures like RT [46,47], we did not observe this behavioural effect. The absence of a negative attention bias is not uncommon in the literature concerning the dot-probe task [12,29,48–50]. The variability in findings suggests that RTs could be influenced by factors such as stimulus duration and threatening intensity [6,48,51]. The intervals between faces and probe presentations also influence RT reliability, with shorter intervals yielding better reliability [52].

Although our ERP results offer direct indices for characterising the distinct temporal profiles of face processing and attentional deployment in individuals with high and low social anxiety, several limitations should be acknowledged. First, the present study focused on healthy adult participants. It is also important to examine whether the electrophysiological responses can be generalised to clinical populations. Second, we did not assess potential comorbidities, such as general trait anxiety, depression or mood disorders, which may also affect face processing and attentional deployment. Therefore, measures of depression and general trait anxiety could be considered and incorporated into data collection and analysis in future work. Third, the cross-sectional nature of the study precludes any causal interpretations. Future research using longitudinal designs could help clarify how emotional expressions influence neural responses in individuals with high social anxiety over time. Furthermore, we presented static facial photographs in a dot-probe task to control for stimulus attributes. However, real-world social interactions are dynamic and multimodal. Future research may also consider employing ecologically valid stimuli, such as films or virtual reality, in both healthy and clinical samples. Finally, future studies could explore interventions that target cognitive avoidance by enhancing face processing in individuals with high social anxiety. Such approaches may enable socially anxious individuals to re-evaluate social information and thereby reduce social-evaluative anxiety.

In summary, this study investigated the temporal dynamics of facial and attentional processing using ERPs when individuals with either high or low social anxiety viewed emotional and neutral faces in a dot-probe task. We found that the HSA group showed reduced facial processing compared with the LSA group. Attention biases towards emotional faces were found only in the HSA group. Our results provide electrophysiological evidence supporting concepts such as self-focus, cognitive avoidance, and the social-evaluation threat principle in social anxiety [8,9,16].

## Supporting information

S1 File Appendix. (DOCX)
