# Supplementary material for: Temporal dynamics of emotional face processing in social anxiety
Source: PLoS One. 2025 Nov 19;20(11):e0337403. doi: 10.1371/journal.pone.0337403 (PMC12629449; doi:10.1371/journal.pone.0337403)
Supplement: S1 File — (DOCX) [file pone.0337403.s001.docx]

**Appendix**

**Participant Profile**

Table 1. Means (standard deviation) and statistics for demographics.

|  | High social anxiety group (*n* = 56) | Low social anxiety group (*n* = 47) | Difference test *(t* and χ*^2^* tests) |
| --- | --- | --- | --- |
| Age* | 19.93 (1.08) | 19.70 (1.38) | *t*(100) = 0.96, *p* = .341 |
| Sex | 59% female (*n* = 33) | 57% female (*n* = 27) | χ*^2^(1)* = 0.02, *p* = .879 |
| SAD | 91.23 (11.60) | 53.13 (9.74) | *t*(101) = 17.85, *p* < .001 |
| FNE | 114.11(13.85) | 76.17 (13.83) | *t*(101) = 13.86, *p* < .001 |
| Accuracy | 95.50% (4.10%) | 95.02% (3.92%) | *t*(101) = 0.60, *p* = .548 |

Note: SAD = Social Avoidance and Distress Scale; FNE = Fear of Negative Evaluation Scale.

* One age data from the LSA group was missing.

**Behavioural Results**

Table 2. Attention bias scores (ms, SD) for each face pair in two groups.

| Face pair | HSA group | LSA group |
| --- | --- | --- |
| Angry-Neutral | 1.20 (21.32) | 3.19 (23.30) |
| Happy-Neutral | 3.90 (18.56) | 1.28 (24.61) |
| Angry-Happy | 1.01 (20.38) | -7.18 (21.16) |

Table 3. ANOVA analysis for accuracy.

| Main effect or interaction | Test |
| --- | --- |
| Group | *F*(1, 101) = 0.004, *p* = .439 |
| Face pair | *F*(3, 303) = 1.02, *p* = .383 |
| Group x Face pair | *F*(3, 303) = 1.04, *p* = .375 |

Note: Mauchly’s test of sphericity: χ²(5) = 4.84, *p* = .436.

Table 4. t-test for attention bias score between groups (ABS).

| Face Pair Condition | Test |
| --- | --- |
| Angry-neutral | *t*(101) = -0.45, *p* = .651 |
| Happy-neutral | *t*(101) = 0.62, *p* = .539 |
| Angry-happy | *t*(101) = 2.00, *p* = .048 |

**ERP Results**

**P1 amplitude**

Table 5. ANOVA analysis for P1 amplitude.

| Main effect or interaction | Test |
| --- | --- |
| Group | *F*(1, 101) = 0.007, *p* = .932 |
| Face pair | *F*(2.69, 303) = 0.75, *p* = .525 |
| Face pair x Group | *F*(3, 303) = 0.61, *p* = .607 |
| Electrode site | *F*(1, 101) = 22.28, *p* < .001 |
| Electrode site x Group | *F*(1, 101) = 0.02, *p* = .884 |
| Face pair x Electrode site | *F*(3, 303) = 0.48, *p* = .697 |
| Face pair x Electrode site x Group | *F*(3, 303) = 0.27, *p* = .846 |

Note: Face pair Mauchly’s test of sphericity: χ²(5) = 17.79, *p* = .003.

Electrode Site x Face pair Mauchly’s test of sphericity: χ²(5) = 4.47, *p* = .484

**N170 amplitude**

Table 6. ANOVA analysis for N170 amplitude.

| Main effect or interaction | Test |
| --- | --- |
| Group | *F*(1, 101) = 4.92, *p* = .029 |
| Face pair | *F*(3, 303) = 0.63, *p* = .595 |
| Face pair x Group | *F*(3, 303) = 0.65, *p* = .586 |
| Electrode site | *F*(1, 101) = 3.91, *p* = .051 |
| Electrode site x Group | *F*(1, 101) = 0.27, *p* = .607 |
| Face pair x Electrode site | *F*(2.70, 303) = 0.12, *p* = .933 |
| Face pair x Electrode site x Group | *F*(3, 303) = 2.76, *p* = .042 |

Note: Face pair Mauchly’s test of sphericity: χ²(5) = 4.56, *p* = .472.

Electrode Site x Face pair Mauchly’s test of sphericity: χ²(5) = 17.03, *p* = .004

**N2pc amplitude**

Table 7. ANOVA analysis for N2pc amplitude in angry-neutral face pair condition.

| Main effect or interaction | Test |
| --- | --- |
| Group | *F*(1, 101) = 0.63, *p* = .430 |
| Electrode site | *F*(1, 101) = 13.06, *p* < .001 |
| Group x Electrode site | *F*(1, 101) = 0.63, *p* = .430 |

Table 8. ANOVA analysis for N2pc amplitude in happy-neutral face pair condition.

| Main effect or interaction | Test |
| --- | --- |
| Group | *F*(1, 101) = 1.01, *p* = .319 |
| Electrode site | *F*(1, 101) = 10.24, *p* = .002 |
| Group x Electrode site | *F*(1, 101) = 0.13, *p* = .718 |

Table 9. ANOVA analysis for N2pc amplitude in angry-happy face pair condition.

| Main effect or interaction | Test |
| --- | --- |
| Group | *F*(1, 101) = 3.89, *p* = .051 |
| Electrode site | *F*(1, 101) = 1.21, *p* = .275 |
| Group x Electrode site | *F*(1, 101) = 0.01, *p* = .981 |

**Correlations between attention bias score and ERPs**

Table 10. Correlations between Attention bias scores and ERP mean amplitudes.

| ERP  Bias score | P1  Angry-Neutral | P1  Happy-Neutral | P1  Angry-Happy | N170  Angry-Neutral | N170  Happy-Neutral | N170  Angry-Happy | N2pc co-ip  Angry-Neutral | N2pc co-ip  Happy-Neutral | N2pc co-ip  Angry-Happy |
| --- | --- | --- | --- | --- | --- | --- | --- | --- | --- |
| Angry-Neutral | 0.06 | -0.05 | 0.07 | -0.08 | -0.13 | -0.09 | .21* | -0.07 | -0.03 |
| Happy-Neutral | 0.14 | 0.09 | 0.17 | -0.07 | -0.04 | -0.04 | -0.16 | 0.03 | 0.06 |
| Angry-Happy | 0.13 | 0.02 | 0.10 | .23* | 0.14 | 0.18 | -0.04 | -0.03 | -0.13 |

*Indicates significant at *p* < .05.
